# Base editing-mediated precision genome engineering in *Toxoplasma gondii*

**DOI:** 10.1371/journal.pntd.0014864

**Published:** 2026-11-13

**Authors:** Fang Liu, Youlan Wu, Zhenghe Chen, Xiang Liu, Li Yu, Jilong Shen, Yuanhong Xu, Jing Yuan, Jiabin Li, Weisheng Cheng

**Affiliations:** 1 Department of Clinical Laboratory, The First Affiliated Hospital of Anhui Medical University, Hefei, Anhui, China; 2 Anhui Province Key Laboratory of Zoonoses, Anhui Medical University, Hefei, Anhui, China; 3 Provincial Key Laboratory of Zoonoses of High Institutions in Anhui, Anhui Medical University, Hefei, Anhui, China; 4 Prenatal Diagnosis Center, Department of Obstetrics and Gynecology, The First Affiliated Hospital of Anhui Medical University, Hefei, Anhui, China; 5 NHC Key Laboratory of Study on Abnormal Gametes and Reproductive Tract, Anhui Medical University, Hefei, Anhui, China; 6 Anhui Province Key Laboratory of Reproductive Disorders and Obstetrics and Gynecology Diseases, Anhui Medical University, Hefei, Anhui, China; 7 Xinjiang Second Medical College, Karamay, Xinjiang, China; 8 Department of Infectious Diseases, The First Affiliated Hospital of Anhui Medical University, Hefei, Anhui, China; 9 Anhui Province Key Laboratory of Infectious Disease, Anhui Medical University, Hefei, Anhui, China; Tokat Gaziosmanpaşa University Faculty of Medicine: Tokat Gaziosmanpasa Universitesi Saglik Bilimleri Fakultesi, TÜRKIYE

## Abstract

Functional genomics in *Toxoplasma gondii* (*T. gondii*), an obligate intracellular parasite of global medical importance, primarily relies on CRISPR-Cas9-mediated gene disruption. However, conventional CRISPR-Cas9 editing relies on generating double-strand breaks (DSBs), which are repaired either by error-prone non-homologous end joining or by homology-directed repair requiring donor templates and time-consuming clone isolation. Here, we established the first two base editor platforms in *T. gondii*: adenine base editors (ABEs) and cytosine base editors (CBEs), which enable precise point mutations via direct base conversion without DSBs or donor templates. We successfully employed ABE and CBE to introduce high-efficiency A > G and C > T conversions at defined genomic loci, respectively. By targeting the critical virulence gene *ROP18*, we demonstrate that ABE-mediated i-Silence (start codon disruption) and CBE-mediated i-STOP (premature stop codon introduction) strategies can efficiently generate functional *T. gondii* null mutants. The resulting base-edited parasite strains exhibited a complete loss of ROP18 protein expression and showed attenuated virulence and proliferation phenotypes equivalent to those of a conventional *ROP18* knockout strain, as validated in both cellular and mouse models. We then conducted a genome-wide analysis predicting that the i-Silence and i-STOP strategies enable targeted inactivation of nearly all genes in the *T. gondii* genome (>99.6% coverage). Since our approach does not require DSBs, it enables researchers to achieve efficient, precise nucleotide substitutions without the risk of chromosomal rearrangements. We propose base editing as a powerful genetic tool in *T. gondii*, paving the way for high-resolution functional pathogenesis studies and the further development of genetically stable attenuated vaccines.

## Author summary

*Toxoplasma gondii* infects a substantial portion of the global population and can cause severe zoonotic disease. CRISPR-Cas9 has greatly facilitated the study of gene function in *Toxoplasma gondii* by efficiently generating gene knockouts. However, for precise point mutations, its dependence on DNA double-strand breaks and donor templates presents limitations. Here, we introduced a more advanced “base editing” system based on CRISPR-Cas9 into *Toxoplasma gondii* for the first time, enabling high-efficiency targeted single-base changes in the genetic code without breaking the DNA. Using ABE-mediated i-Silence and CBE-mediated i-STOP strategies, we precisely inactivated a vital virulence gene, *ROP18*, either by disrupting its start signal or by introducing an early stop signal, as a proof-of-concept study. The edited parasite’s virulence was significantly reduced, matching the effects of complete gene deletion but achieved with greater precision and safety. Our study has proven that novel base editing is a versatile and accurate tool for studying the biology of *Toxoplasma gondii*, furthermore the i-Silence and i-STOP strategies enable a large-scale functional genetic screen proposal.

## Introduction

*Toxoplasma gondii* (*T. gondii*) is a widespread obligate intracellular apicomplexan parasite, posing severe risks to immunocompromised individuals and fetuses via congenital transmission [1,2]. Despite *T. gondii’s* significant impact on human and animal health, with an estimated global seroprevalence ranging from 30-50% depending on geographic region, the molecular mechanisms underlying its pathogenesis, immune evasion, and stage conversion remain incompletely understood [3,4].

Genetic manipulation in *T. gondii* has evolved considerably over the past two decades. Early methods relied on homologous recombination with positive-negative selection markers, which were labor-intensive and time-consuming [5]. The advent of CRISPR-Cas9 technology revolutionized *T. gondii* genetics, enabling rapid gene knockout via non-homologous end joining (NHEJ) or homology-directed repair (HDR) [6]. However, this technology still has limitations, including double-strand breaks (DSBs), which can trigger unpredictable indels, chromosomal rearrangements, large deletions, and cell toxicity [7]. Moreover, HDR-mediated precise point mutations in *T. gondii* still require exogenous donor templates and exhibit variable efficiency [8]. These limitations pose challenges for applications requiring precise nucleotide changes, such as fine-tuning gene function at the single-nucleotide level, modeling pathogenic single-nucleotide polymorphisms (SNPs), or validating drug targets by introducing resistance mutations [9].

Base editing (BE) represents a transformative advance, relying on fusion proteins that combine a catalytically impaired Cas9 (nickase Cas9) with a deaminase enzyme. This facilitates specific types of base conversions (C > T or A > G) without inducing DSBs. Cytosine base editors (CBEs) induce the conversion of cytosine to thymine (C•G to T•A) through cytidine deaminase activity [10], and adenosine base editors (ABEs) enable the conversion of adenine to guanine (A•T to G•C) through engineered adenosine deaminases [11]. These BEs have found wide-ranging applications in various fields, including bacteria [12], models of human disease [13], plant variety enhancement [14], and, more recently, *Leishmania spp* [15,16]. Furthermore, editing tools have been extensively studied for gene therapy of Mendelian disorders, including β-thalassemia, Stargardt disease, and spinal muscular atrophy [17–20]. Based on BEs, researchers have also developed novel gene inactivation strategies. For instance, CBE-mediated CRISPR-STOP [21] and i-STOP [22] introduce premature stop codons (converting CAA/CAG glutamine codons or CGA arginine codons to TAA/TAG/TGA stop codons). Meanwhile, ABE-based i-Silence disrupts start codons (converting ATG methionine to GTG valine or ACG threonine) [23]. Although these strategies have been widely implemented in various organisms, including bacteria and human cells, their application in *T. gondii* remains unexplored. Therefore, we hypothesize that base editing may produce specific genetic modifications, offering a distinct advantage over conventional CRISPR-Cas9 technology in *T. gondii*.

In this study, we constructed optimized ABE and CBE plasmids specifically for *T. gondii* and established the first functional base-editing platform for this parasite. We then demonstrate that both i-Silence and i-STOP strategies effectively inactivate key virulence factor *ROP18* as a proof-of-concept study, yielding phenotypes indistinguishable from those of traditional knockouts across multiple assay systems. A comprehensive genome-wide map of targetable sites is first presented, revealing that >99% of *T. gondii* genes are amenable to this strategy. Overall, we adapt base-editing strategies to introduce specific, well-defined point mutations in *T. gondii*, providing a robust, scalable and precise tool for functional genomics, drug target validation, and vaccine development, while addressing critical limitations of existing genetic manipulation methods.

## Materials and methods

### Ethics statement

Animal experiments were approved by the Institutional Review Board of Anhui Medical University (No. LLSC20200036). The animals were housed under standard laboratory conditions, following the guidelines provided by the Chinese National Institute of Health for the care and use of laboratory animals.

### Cell and parasite culture

Human Foreskin Fibroblasts (HFF; ATCC SCRC-1041) were cultured in high-glucose Dulbecco’s Modified Eagle’s Medium (DMEM, VivaCell, C3113), supplemented with 15% fetal bovine serum (FBS, Biowest, S711-001S), 2 mM L-glutamine, 100 U/mL penicillin, and 100 μg/mL streptomycin. Neuro-2a neuroblastoma cells (ATCC CCL-131) were cultured in Eagle’s Minimum Essential Medium (Gibco, C11095500BT), supplemented with 10% FBS, 100 U/mL penicillin, and 100 μg/mL streptomycin. Cells were passaged every 3–4 days upon reaching 80–90% confluence using 0.25% trypsin-EDTA. The ME49 strain of *T. gondii* was maintained in HFF monolayers at 37°C under 5% CO_2_ in a humidified incubator. Parasites were harvested by syringe passage (25-gauge needle), and viability was assessed by trypan blue exclusion; only preparations with >95% viability were used for experiments.

All cultures were maintained at 37°C under 5% CO_2_, and mycoplasma contamination was tested monthly using MycoBlue Mycoplasma Detector (Vazyme, D101).

### Plasmid construction

To generate the *Tg*-ABE and *Tg*-CBE plasmid system, the ABE8e and YE1-BE3 expression cassettes were synthesized (GENEWIZ) according to the plasmid ABE8e (addgene#138489) and CMV-YE1-BE3-FNLS-CMV-mCherry (addgene#154005), then cloned into a modified pUC19 backbone containing: (1) the *T. gondii* U6 promoter (450 bp upstream of U6 snRNA transcription start site) for sgRNA expression, (2) the *T. gondii* β-tubulin (TUB1) promoter (1.2 kb) for expression of BE and the dihydrofolate reductase thymidylate synthase (DHFR-TS) selection cassette (pyrimethamine resistance), and (3) the 3’ UTR of *T. gondii* DHFR-TS for transcript stability. To express a gRNA, the oligonucleotide duplexes were cloned into the *Aar* I restriction sites of the *Tg*-ABE or *Tg*-CBE plasmid.

For the *ROP18*-KO targeting assay, CRISPR-CAS9 plasmid targeting *ROP18* (pSAG1::CAS9-U6::sg*ROP18*) was engineered from the pSAG1::CAS9-U6::sg*UPRT* backbone (Addgene #54467) using the Q5 Site-Directed Mutagenesis Kit (New England Biolabs, E0554) to substitute the *UPRT*-targeting gRNA with *ROP18*-specific sgRNAs. To generate a plasmid for inserting DHFR* into the *ROP18* gene (p*ROP18*::DHFR-D), 5’ (820-bp) and 3’ (850-bp) regions flanking the sg*ROP18* target region were used to surround the DHFR* cassette and cloned into pUC19 using ClonExpress MultiS One Step Cloning Kit (Vazyme, C113).

All sgRNAs and primer sequences used in this study are listed in S1 and S2 Tables.

### *T. gondii* transfection, selection, and subcloning

For the base-editing assay, freshly lysed ME49 tachyzoites were purified by filtration and counted with a hemocytometer. For each transfection, 1 × 10^7^ tachyzoites were resuspended in Cytomix buffer (120 mM KCl, 0.15 mM CaCl_2_, 10 mM K_2_HPO_4_/KH_2_PO_4_, 25 mM HEPES, 2 mM EGTA, 5 mM MgCl_2_, pH 7.6). Plasmid DNA was added to the parasite suspension together with freshly prepared ATP and glutathione (final concentrations of 2 mM and 5 mM), and mixed gently. For the base-editing assay, a total of 50 μg of purified circular plasmid DNA was mixed with freshly harvested parasites and electroporated under the conditions described. Electroporation was performed using a Bio-Rad Gene Pulser Xcell electroporation system (Bio-Rad, 1652660) with a 4 mm gap cuvette (Bio-Rad, 1652081) at 1.5 kV, 25 μF, and 200 Ω, yielding a time constant of 4.5-5.0 ms. Post-electroporation parasites were immediately inoculated onto fresh HFF monolayers in T25 flasks and allowed to recover for 24 hours in complete medium without drug selection. Drug selection was initiated 48 hours post-transfection using 3 μM pyrimethamine (Sigma-Aldrich, 46706) in complete medium for 48 hours, and resistant populations were expanded for 7–10 days. Single clones were obtained by limiting dilution in 96-well plates containing HFF monolayers. Parasites were serially diluted to an average of one parasite per two wells. Genomic DNA was extracted from positive wells using the DNA Isolation Kit (Vazyme, DC102), and clones were verified by PCR amplification of the target region followed by Sanger sequencing (GENEWIZ).

To generate *ROP18*-deficient ME49 parasites, we followed a previously established protocol [6]. Briefly, the sg*ROP18* CRISPR plasmid (pSAG1::CAS9-U6::sg*ROP18*) was co-delivered with a repair template (p*ROP18*::DHFR-D) carrying a pyrimethamine-resistant DHFR cassette flanked by *ROP18* homology arms, at a mass ratio of 5:1. A total of 15 μg of the linearized donor plasmid was used for electroporation to mediate homology-directed integration. Transfected populations were subjected to drug selection with 3 μM pyrimethamine for 7–8 days to isolate stable, resistant clones. These clones were initially screened by PCR to verify the correct homologous recombination at the *ROP18* locus.

Subsequently, sequencing-positive candidates or PCR-positive candidates were validated by Western blotting to confirm complete ablation of ROP18 protein expression.

The sgRNAs and primers used in this study are listed in S1 and S2 Tables.

### Targeted sequencing

Genomic DNA was isolated using the DNA Isolation Kit (Vazyme, DC102). Regions of interest were amplified using Phanta Max Master Mix (Vazyme, P525). Purified PCR products were sequenced on an ABI 3730xl DNA Analyzer. Editing efficiencies were quantified using the EditR software [24]. Peak heights from Sanger sequencing chromatograms were analyzed to calculate the percentage of base conversion.

### Amplicon deep sequencing for on-target editing efficiency and off-target analysis

For off‑target assessment, potential off‑target sites were first predicted using the CRISPOR [25] for each sgRNA. Target regions flanking each sgRNA recognition site were amplified by PCR using locus‑specific primers appended with Illumina adapter sequences. A second‑round PCR was then performed to incorporate sample‑specific index tags for multiplexing. All PCR reactions were carried out with Phanta Max Master Mix (Vazyme, P525) to ensure high‑fidelity amplification. The indexed amplicon libraries were purified, quantified, pooled in equimolar amounts, and subjected to paired‑end sequencing (150 bp) on an Illumina NovaSeq 6000 platform by ANOROAD. For the *ROP18*-ABE sample, the amplicons were subjected to Oxford Nanopore Technology (ONT) library preparation and sequenced by Sangon Biotech using the KBSeq third-generation sequencing service on a PromethION 2 Solo platform. Sequencing reads were analyzed using CRISPResso2 [26]. Editing efficiencies and mutation profiles were quantified from amplicon sequencing data using CRISPResso2: quantification window center was set to -3, window size to 1 bp, minimum alignment score to 60, minimum average read quality to 0 (Phred33), minimum per-base quality/N threshold to 0, and 15 bp were excluded from both the left and right ends of the amplicon. For on‑target analysis, editing efficiency was calculated as the proportion of reads at the target base position relative to total reads. For off‑target analysis, the edited samples were compared against the control group to identify potential off‑target mutations, including both single-nucleotide variants and indels, at each predicted off‑target locus.

The corresponding genomic regions were amplified using primer pairs listed in the S2 Table (primers) and S3 Tables (off‑target loci).

### Plaque assays

Freshly harvested tachyzoites (1 × 10^3^ per strain, quantified via hemocytometer using trypan blue exclusion) were inoculated onto HFF monolayers in six-well plates in triplicate. Cultures were maintained at 37℃ in 5% CO_2_ for 10 days, with medium replaced on day 5. Subsequently, cells were fixed with 4% paraformaldehyde in PBS for 20 min at room temperature, washed three times with PBS, and stained with 0.1% crystal violet (Sigma-Aldrich, C6158) for 30 min.

### *T. gondii* intracellular multiplication assays

HFF cells were seeded on glass coverslips in 24-well plates and infected with tachyzoites at an MOI of 3. After 24 hours of infection at 37°C with 5% CO_2_, cells were fixed with ice-cold methanol for 10 min and stained with Giemsa (Beyotime, C0131) diluted 1:10 in distilled water for 30 min. Coverslips were mounted on glass slides with Permount mounting medium (Fisher Scientific, SP15–100). The number of intracellular tachyzoites per parasitophorous vacuole was counted under a light microscope, and a minimum of 200 randomly selected host cells were counted per strain across three independent experiments.

### Western blotting

Parasites were lysed in RIPA buffer (Cell Signaling Technology, 9806) supplemented with protease inhibitors (Roche, 11836153001) and phosphatase inhibitors (Sigma-Aldrich, P5726, P0044) on ice for 30 min. Lysates were centrifuged at 14,000 × g for 15 min at 4°C, and supernatants were collected. Protein concentrations were determined using the BCA Protein Assay Kit (Thermo Fisher, 23225). 30 μg of total protein was loaded per lane, separated on 10% SDS-PAGE gels, and transferred to PVDF membranes (GE Healthcare, 10600023). Membranes were blocked with 5% non-fat milk in TBST (Tris-buffered saline with 0.1% Tween-20) for 1 hour at room temperature and incubated overnight at 4°C with primary antibodies: anti-Cas9 (Diagenode, C15310258, 1:1000), anti-ROP18 (in-house prepared, 1:1000) [27], and anti-Profilin (loading control, 1:2000). After washing, membranes were incubated with HRP-conjugated secondary antibody (1:5000) for 1 hour at room temperature. Signals were visualized using Clarity Western ECL Substrate (Bio-Rad) and quantified using Image Lab software.

### Virulence in laboratory mice

Six-week-old female C57BL/6 mice were divided into four groups (5 mice per group): WT, *ROP18*-KO, *ROP18*-i-Silence, *ROP18*-i-STOP. Each mouse was injected intraperitoneally with 100,000 tachyzoites, and survival was monitored daily for 30 days.

### Mouse euthanasia, brain collection, and cyst enumeration by brain squash

At 30 days post‑infection, the surviving mice from each group were euthanized by cervical dislocation, and whole brains were aseptically removed. Each brain was bisected along the midsagittal plane. One hemisphere was weighed and homogenized in 2 mL of distilled water using a tissue grinder. Three aliquots of 25 μL each were taken from the homogenate and placed on glass slides, then examined under a light microscope at 200× or 400 × magnification. Tissue cysts, identified by their characteristic round to oval shape, refractile cyst wall, and densely packed intracyptic bradyzoites, were counted in each aliquot. The average cyst number per 25‑μL sample was calculated, and the total cyst number per hemisphere was extrapolated based on the total homogenate volume (2 mL). The final value was doubled to estimate cysts per whole brain.

### Real-time quantitative PCR detection of parasite burden in brain tissues

Genomic DNA was extracted from the brain tissues of parasite-infected mice using the TIANamp Blood and Tissue DNA Kit (Tiangen, DP304) according to the manufacturer’s instructions. Parasite burden was determined by qPCR amplification of the *TgB1* gene using corresponding genomic DNA samples. The values were normalized to the genomic copy number of the mouse *Gapdh* gene in each sample. Real-time qPCR was performed using the ChamQ Universal SYBR qPCR Master Mix (Q711, Vazyme, China) according to the manufacturer’s instructions, and reactions were run on the Roche LC480II system. The threshold cycle (Ct) values were averaged for each sample. Relative quantification of *TgB1* expression was performed using the 2^‑ΔΔCt^ method.

### Flow cytometry analysis

Neuro-2a cells were seeded in 6-well plates (5 × 10^5^ cells/well) and infected with tachyzoites (MOI = 3) for 24 hours. Cells were harvested by trypsinization (0.25% trypsin-EDTA, 5 min at 37°C), washed twice with cold PBS, and resuspended in 100 μL of 1 × binding buffer. Cells were stained with the Annexin V-FITC/PI Apoptosis Detection Kit (Yeasen, 40302ES) according to the manufacturer’s instructions: 5 μL Annexin V-FITC and 5 μL PI were added, and samples were incubated for 15 minutes in the dark at room temperature. After incubation, 400 μL of 1 × binding buffer was added, and samples were analyzed immediately. At least 10,000 events per sample were acquired on a BD FACSCanto II flow cytometer (BD Biosciences) and analyzed with FlowJo (FlowJo, LLC).

### Genome-wide i-STOP and i-Silence target analysis

The *T. gondii* ME49 genome (GCF_000006565.2_TGA4_genomic.gtf) was used for i-STOP and i-Silence analysis. Only protein-coding genes located on chromosome-level assembled sequences and have a start and STOP codon, no internal STOP codons, and a sequence length evenly divisible by three were retained. Potential i-STOP target sites (CAA, CAG, CGA, TGG codons convertible to TAA, TAG, TGA stop codons) and i-Silence target sites (ATG start codons convertible to GTG, ACG) were identified within the editing window of valid protospacer-adjacent motif (PAM) sequences (NG, NGA, NGG, NNNRRT, NGAG, NNGRRT, NGCG). The iSTOP R package (https://github.com/CicciaLab/iSTOP) was used to identify i-STOP targetable sites. An in-house Python script was used to identify i-Silence targetable sites. It read editor-specific parameters from a configuration file and evaluated 7 ABE variants with their specific PAM types, editing window positions, and gRNA lengths: ABE7.10 (NGG, 4–8, 20 nt), ABESa (NNGRRT, 6–12, 21 nt), Sa(KKH)-ABE (NNNRRT, 8–13, 21 nt), VQR-ABE (NGA, 4–8, 20 nt), VRER-ABE (NGCG, 4–8, 20 nt), EQR-ABE (NGAG, 4–8, 20 nt), and ABE-NG (NG, 4–8, 20 nt). The core selection criterion was to preferentially select gRNAs containing only a single ‘A’ within the defined editing window, thereby minimizing bystander editing.

### Statistical analysis

All statistical analyses were performed using GraphPad Prism 9.0. Data are presented as mean ± SD. Differences between the two groups were assessed using a two-tailed Student’s *t*-test. Comparisons among multiple groups were performed using One-way ANOVA followed by Tukey’s post-hoc test. Survival curves were analyzed using the Log-rank (Mantel-Cox) test. Pairwise comparisons of normalized off-target editing efficiency between groups at each predicted off-target locus were performed using multiple two-tailed Welch’s unpaired t-tests. The Benjamini–Hochberg false discovery rate method was applied to correct for multiple testing, and the resulting q-values were used to evaluate statistical significance. A q-value < 0.05 was defined as statistically significant. A P-value < 0.05 was considered statistically significant.

## Results

### BE system facilitates base conversion in *T. gondii*

To adapt the BE system to *T. gondii*, we constructed plasmids expressing ABE (ABE8e) and CBE (YE1-BE3), respectively, with species-specific promoters (*Tg*U6 for sgRNA, *Tg*TUB1 for editor expression) and appropriate selection markers (DHFR-TS for pyrimethamine resistance) (Fig 1A). The TUB1 promoter was selected based on previous studies demonstrating strong, constitutive expression in tachyzoites [28], while the U6 promoter ensures efficient sgRNA transcription [8]. Immunoblot analysis confirmed robust expression of the BE proteins in tachyzoites, with peak expression observed at 48 hours post-electroporation and sustained expression for up to 7 days (Fig 1B).

**Fig 1:**
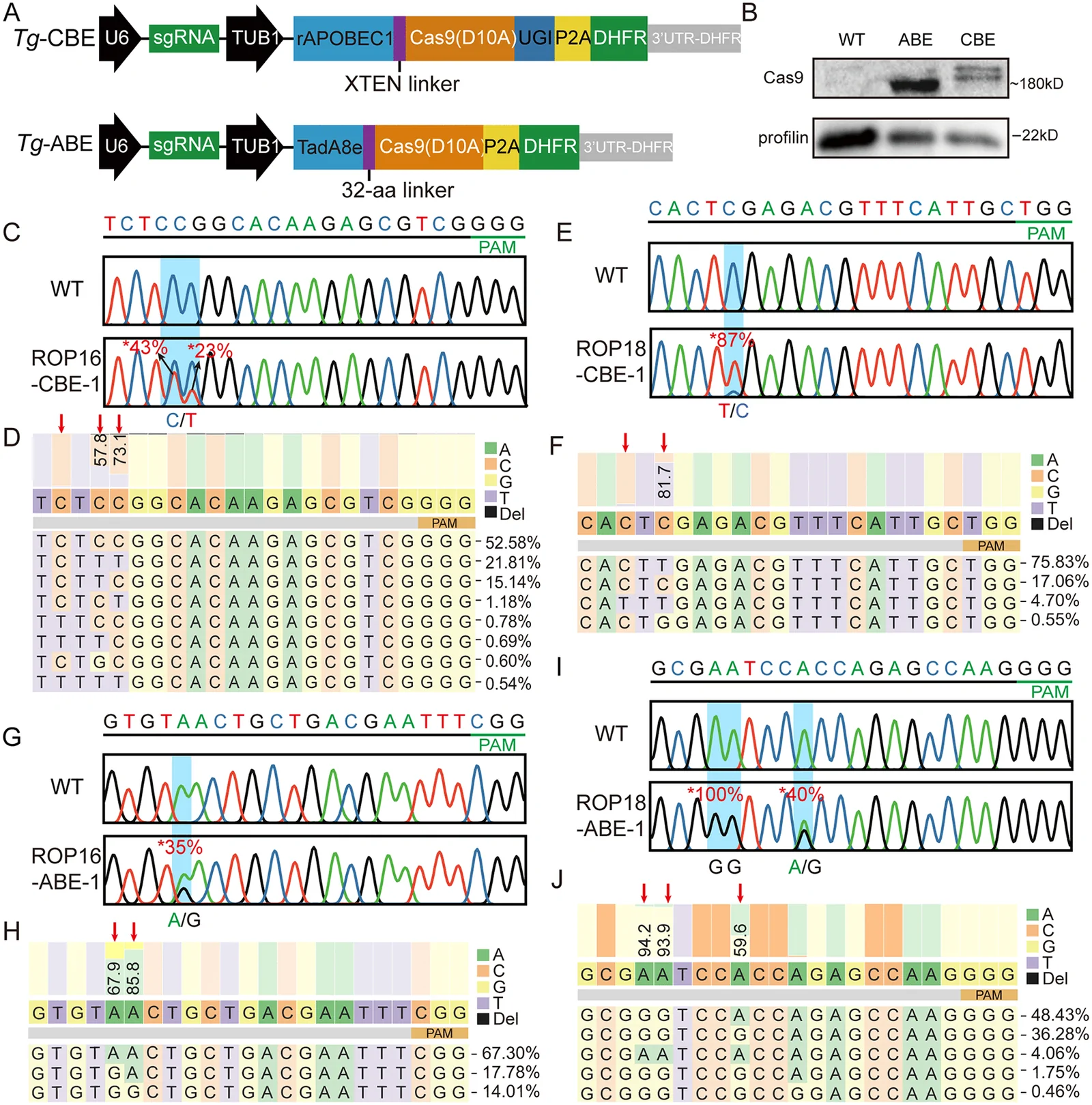
The BE system facilitates base conversion in *T. gondii.* (A) Diagram of plasmids expressing CBE (Top) and ABE (Bottom) systems in *T. gondii.* (B) Expression of base editor proteins in *T. gondii* at 48 h post‑transfection, as assessed by immunoblotting with profilin as a loading control. (C-J) Quantitative analysis of base editing efficiency at *ROP16* and *ROP18* loci assessed by Sanger sequencing **(C, E, G, I)** and amplicon deep sequencing **(D, F, H, J)**.

To evaluate editing efficiency, we targeted *ROP16* and *ROP18* loci with multiple sgRNAs, respectively. Given the narrow editing window of base editors (nucleotides 4–8 of the protospacer), editing efficiency varied significantly with sgRNA positioning, reaching its optimum within this window [10,29]. Sanger sequencing analysis initially revealed that the CBE system achieved conversion efficiencies ranging from 23% to 87% across different target sites, while the ABE system showed efficiencies ranging from 35% to 100%. To precisely quantify editing outcomes and resolve potential mixed-editing populations, we performed high‑throughput amplicon deep sequencing on all target loci. Although the deep sequencing data largely confirmed the overall editing patterns observed by Sanger sequencing, the measured efficiencies showed slightly narrower ranges: 26.9%–81.7% for CBE and 32.1%–94.2% for ABE (Fig 1C–1J and S4 Table). This discrepancy likely reflects the inherent limitations of Sanger‑based estimation—particularly the inability to resolve mixed-editing populations and distinguish bystander mutations—whereas deep sequencing provides more accurate quantification. Notably, the high‑throughput approach also enabled precise detection of bystander editing events, which occurred at extremely low frequencies. Taken together, these data confirm that both base‑editing systems are highly efficient at the targeted loci in *T. gondii*, while deep sequencing offers a more refined and reliable measurement of editing outcomes.

### BE-mediated *ROP18* gene disruption

CBE-mediated CRISPR-STOP [21] and i-STOP [22] strategies were employed to introduce premature stop codons by converting specific sense codons, thereby leading to translational termination. Meanwhile, the i-Silence [23] strategy was used to disrupt the start codon, thereby inactivating the gene. To evaluate the effectiveness of the i-Silence and i-STOP strategies against *T. gondii*, we chose *ROP18* as a target, a key virulence factor in the rhoptry protein kinase family. We generated three mutant strains: a conventional knockout (ME49-*ROP18*-KO) using CRISPR-Cas9-mediated HDR (Fig 2A and 2B), an i-STOP strain (ME49-*ROP18*-i-STOP) with CAG > TAG premature stop codon, and an i-Silence strain (ME49-*ROP18*-i-Silence) with ATG > GTG start codon disruption. Sanger sequencing of the monoclonal lines revealed a single clean peak, confirming targeted editing (Fig 2C and 2D). Deep sequencing showed a targeted substitution frequency of 99.32% for the i-Silence clone and a frequency of 98.76% for the i-STOP clone, with all displayed sequences edited within the target region. An additional 2.42% of reads in the i-STOP clone corresponded to natural variations outside the target region (also present in wild-type controls), while the remaining undisplayed reads (~0.72% for i-Silence and ~1.24% for i-STOP) were attributable to background errors from sequencing and PCR [30]. No unedited target sequences were detected in either clone. Given that *T. gondii* tachyzoites are haploid and both clones were derived from a single parasite, these results demonstrate complete clonal editing at the target loci (Fig 2E and 2F). Western blotting showed no detectable ROP18 protein band upon visual inspection of blots from all three mutant strains. Band densitometry only captured low-level background noise, leading to a significantly reduced measured signal compared with wild-type controls (Fig 2G).

**Fig 2:**
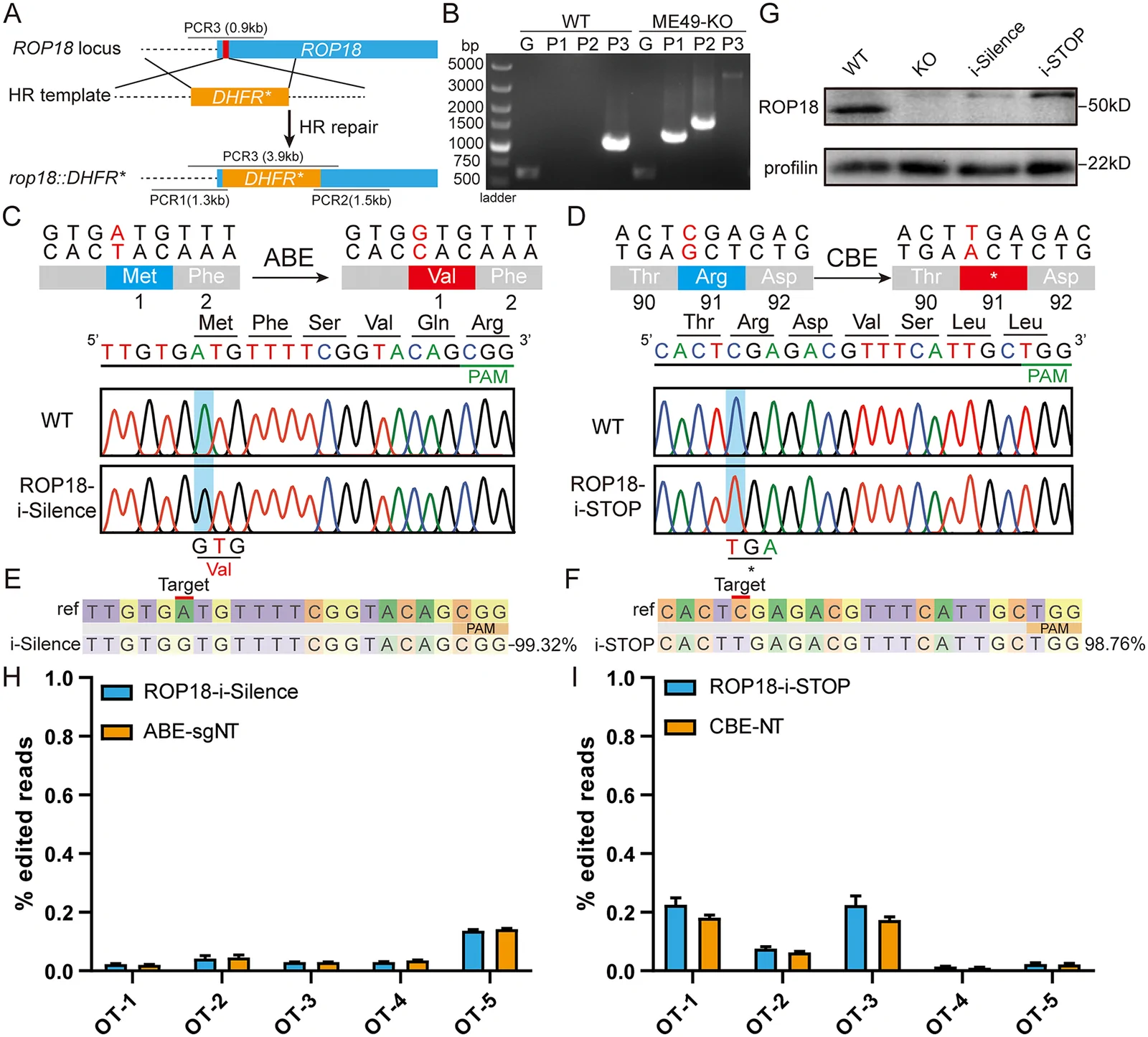
BE-mediated *ROP18* gene disruption. (A) Schematic diagram for knocking out the *ROP18* locus. (B) Diagnostic PCR showing integration of the DHFR* cassette in ME49-*ROP18*-KO clone. G: *GRA1* promoter positive control. P1: 5’ integration. P2: 3’ integration. P3: DHFR integration. (C-D) Quantitative analysis of base editing efficiency ABE induced disruption of ROP18 at *ROP18-i-Silence* loci through Sanger sequencing **(C)** and amplicon deep sequencing **(D)**. (E-F) Quantitative analysis of base editing efficiency CBE induced disruption of ROP18 at *ROP18-i-STOP* loci through Sanger sequencing **(E)** and amplicon deep sequencing **(F)**. (G) ROP18 expression assessed by immunoblotting with profilin as a loading control. (H-I) Off-target editing rates at five predicted sites for ABE **(H)** and CBE **(I)**. Group comparisons for off-target editing efficiency at each off-target site were conducted via multiple two-tailed Welch’s t-tests, followed by Benjamini–Hochberg FDR multiple testing correction. Statistical significance was determined at q < 0.05.

To evaluate the specificity of ABE and CBE, we performed amplicon deep sequencing at the top five highest-scoring computationally predicted off‑target loci (CRISPOR) for each editor. No off-target editing was detected at any of these loci, at a median read depth of 551,363 (limit of detection 0.0028%) (Fig 2H -2I and S4 Table). Collectively, these results demonstrate that neither editing system induced detectable off‑target alterations at the evaluated loci within the limits of our detection.

### BE‑mediated ROP18 disruption attenuates *T. gondii* virulence *in vitro* and *in vivo*

Having established the high efficiency and specificity of base editing at the target loci, we next sought to determine whether disruption of ROP18 via ABE‑mediated i‑Silence or CBE‑mediated i‑STOP could recapitulate the attenuated phenotype of the conventional *ROP18* knockout strain. To this end, we systematically evaluated the virulence of these mutant strains through a series of *in vitro* and *in vivo* assays In murine virulence assays, mice infected with any of the three mutant strains exhibited 100% survival over 30 day monitoring period. In contrast, the WT-infected group exhibited a stepwise decrease in survival rate starting from day 8, with a final survival rate of 40% at the end of the experiment (Fig 3A). We enumerated brain cysts by microscopy and then quantified parasite burden by qPCR targeting the *TgB1* gene. Both analyses revealed significant differences among the four groups (*P* < 0.0001). Cyst counts were dramatically reduced in the KO, i‑Silence, and i‑STOP groups compared to WT controls (Fig 3B), which was further corroborated by qPCR analysis showing a consistent significant difference across the groups (*P* < 0.0001) (Fig 3C). Intracellular multiplication assays revealed that the WT strain showed robust proliferation, with 41.17% of vacuoles containing 8 or ≥16 parasites. In contrast, KO, i-Silence, and i-STOP strains exhibited significantly impaired replication. Across all three mutant strains, at least 41.43% of vacuoles containing ≤2 parasites, whereas vacuoles containing ≥16 parasites were almost absent. No significant difference was observed among the three genetically modified strains (Fig 3D). Plaque assays further confirmed the attenuation of the mutant strains, which formed significantly smaller plaques than the WT controls (*P* < 0.0001) (Fig 3E and 3F). Regarding neuroapoptosis, a known function of ROP18, mediated through phosphorylation of host IRG proteins and modulation of NF-κB signaling, co-culture experiments with Neuro-2a cells demonstrated that all three mutant strains induced significantly lower levels of apoptosis than WT (*P* < 0.001). Flow cytometry analysis showed that WT infection induced 43.3% ± 2.7% apoptotic cells, whereas mutant strains induced only 23–26% apoptosis, with no statistically significant difference among the KO, i-Silence, and i-STOP groups (Fig 3G and 3H). These data confirm that base-editing-mediated gene inactivation via i-Silence or i-STOP yields phenotypic outcomes equivalent to those of traditional gene knockout, validating the reliability and precision of this new platform for functional genomics applications.

**Fig 3:**
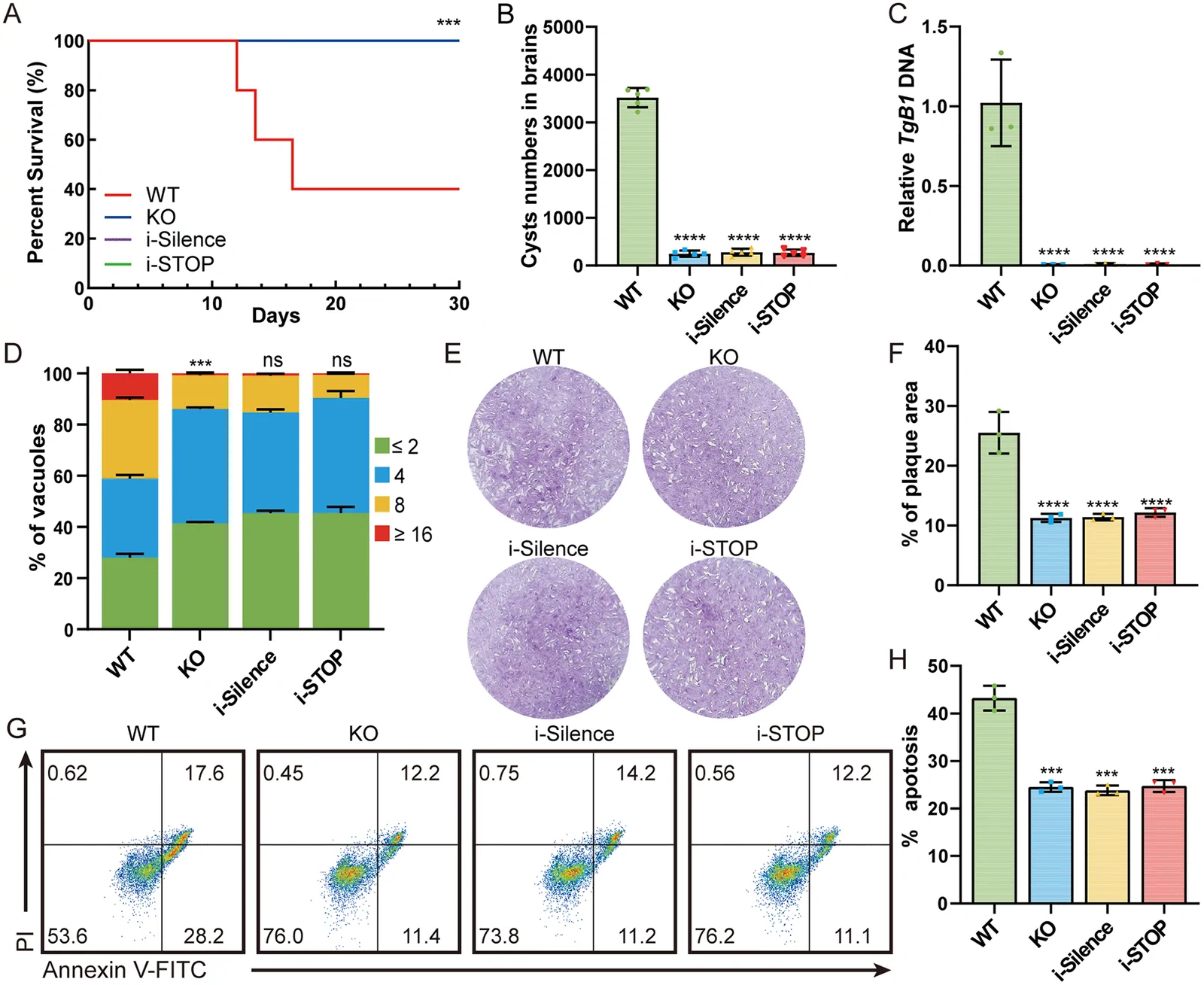
Base editor‑mediated ROP18 disruption attenuates *T. gondii* virulence *in vitro* and *in vivo.* (A) Survival curves of different strains in mice (n = 5). (B) Total cyst numbers per whole brain (n = 5) counted by light microscopy from brain homogenates. (C) Parasite genomic copy number/burden measured by qPCR targeting *TgB1*, with Gapdh as the endogenous reference and relative quantification performed using the 2^−ΔΔCt^ method (n = 3). (D) Intracellular multiplication assays. (E-F) Plaque assay of different ME49 strains. Representative plaque images **(E)** and quantification of plaque area as a percentage of well area **(F)**. (G-H) Apoptosis of infected Neuro-2a cells was determined by Flow cytometry **(G)**, quantification of apoptosis rate**(H)**. *** represents *P* < 0.001, **** represent *P* < 0.0001. ns represents not significant (one-way ANOVA).

### Comprehensive detection of i-STOP and i-Silence Targets in *T. gondii*

To assess the generalizability of our platform, we performed a comprehensive genome-wide scan for targetable sites by i-STOP and i-Silence strategy across 8,154 ORFs of the *T. gondii* genome (Fig 4A and 4F). Our analysis identified that about 50% of the genes in the *T. gondii* genome contain a minimum of 34 i-STOP targets, with over 80% of peptides targetable within the first 10% of their sequences (Fig 4B and S5 Table). This genome-wide analysis revealed that 87.58% of the CAA, CAG, CGA, and TGG codons in the *T. gondii* reference genome can be targeted by i-STOP, thus allowing the possibility to precisely convert 376,123 codons into STOP codons (Fig 4C). Remarkably, in 8,151 out of 8,154 (99.96%) protein-coding transcripts can be targeted by i-STOPs (Fig 4C and 4D). i-STOPs targets of all ORFs were listed in S5 Table. The centralized distribution of targeting sites at the protein level demonstrated the broad applicability of the CBE strategy to the *T. gondii* genome, with only about 0.04% of proteins not targeted for editing (Fig 4E). Seven different PAM sequences were used to assess i-Silence’s potential for genome-wide targeting of the *T. gondii* gene. i-Silence could target 7,998 genes (S6 Table). In comparison, i-STOP could target 8,151 genes, covering approximately 98.07% and 99.96% of the *T. gondii* genome, respectively, with i-STOP marginally surpassing i-Silence in gene coverage (Fig 4G). As visualized in a Venn diagram (Fig 4H), a total of 7995 genes can be targeted by both strategies. Meanwhile, 3 genes can be targeted only by i-Silence, and 157 genes can be targeted only by i-STOP. These results indicate that the i-Silence strategy complements i-STOP for achieving genome-wide targeting.

**Fig 4:**
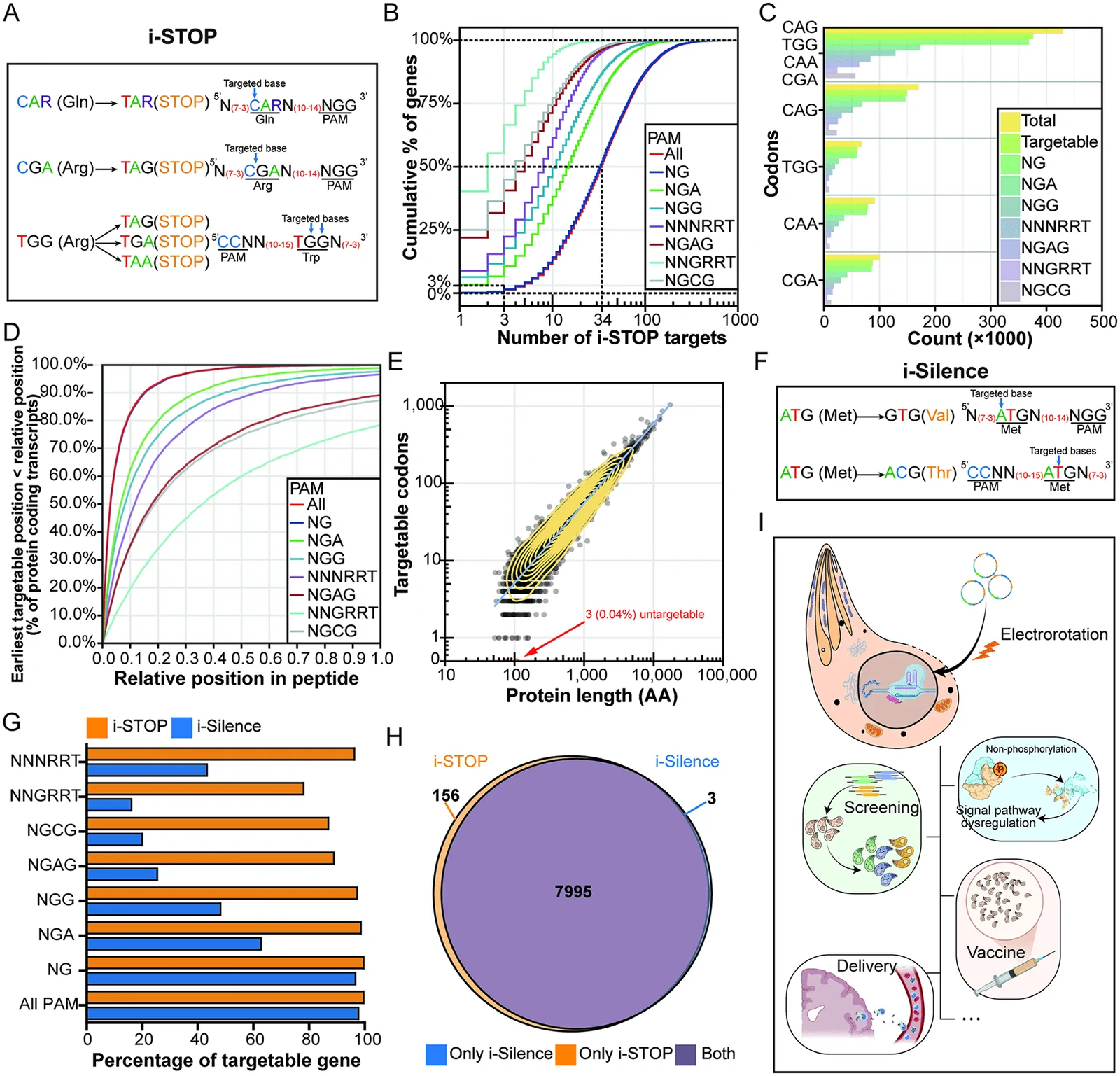
Comprehensive detection of i-STOP and i-Silence targets in *T. gondii.* (A) Schematic diagram of nonsense mutation realized by CBE. (B) The cumulative distribution of i-STOPs per gene is presented, with the number of i-STOPs for an average ORF (50%) in the *T. gondii* genome represented by a dotted line. (C) Number of CAG, TGG, CAA, and CGA codons in the *T. gondii* genome targetable by i-STOP. (D) Relative position of the earliest i-STOP codon targetable in a peptide with distinct PAM specificities. (E) Number of i-STOP targetable codons in proteins of different lengths. (F) Schematic diagram of gene silence achieved by ABE. (G) Percentage of i-Silence and i-STOP targetable *T. gondii* coding genes. (H) A Venn diagram summarizes *T. gondii* coding genes that are (not) targetable by the i-STOP or i-Silence strategy. (I) Prospects for the application of BE in *T. gondii.*

## Discussion

In this study, we present the first functional application of adenine- and cytosine-base-editing systems in *T. gondii*, marking a significant paradigm shift in the genetic manipulation toolkit for this important obligate intracellular parasite. By validating these tools against the virulence gene *ROP18*, we demonstrate that these editors enable precise gene inactivation through two distinct strategies—i-STOP and i-Silence —based on ABE and CBE, respectively. Our genome-wide analysis confirms the broad applicability of i-STOP and i-Silence strategies, revealing that nearly all *T. gondii* coding genes are targetable. This comprehensive coverage enables scalable functional genomic screens to systematically identify novel therapeutic targets or genes essential for the parasite’s complex life cycle.

Crucially, unlike conventional CRISPR-Cas9-mediated knockout, which relies on NHEJ or HDR [31], our approach enables precise, scarless nucleotide transitions (A•T to G•C or C•G to T•A) without inducing DNA DSBs in *T. gondii*. By minimizing the risk of chromosomal rearrangements and large deletions, this strategy offers a critical safety advantage for generating strains intended for vaccine development or therapeutic applications. However, the base-editing efficacy was also shown to be transfection-efficiency-dependent and to vary widely among genes and selected sgRNA sequences. Another worth highlighting is that base editing technically truncates single-nucleotide changes, and ORFs are not entirely replaced [32]. It is unknown whether mutant mRNAs are quickly degraded or have any additional effects on the *T. gondii*, except for the verified *ROP18* gene. Furthermore, the constrained editing window and the potential for bystander edits pose substantial hurdles to BE progress [33].

The capacity of ABE and CBE to generate precise, scarless knockouts, exemplified by ROP18 inactivation, is ideal for developing genetically live-attenuated parasites for vaccine use. Traditional auxotrophic or knockout mutants may revert through compensatory mutations or suffer from genomic instability during long-term culture. In contrast, i-STOP or i-Silence strains carry stable, irreversible point mutations that prevent reversion to virulence while preserving the majority of the proteome for immune recognition. This is particularly important for *T. gondii* vaccine development, as no effective human vaccine currently exists despite the substantial disease burden. The only commercially licensed anti-*Toxoplasma* vaccine to date is Toxovax (Merck & Co., Inc.), a live-attenuated preparation of the S48 strain of *T. gondii*. Its utility is confined to the veterinary domain, specifically for immunizing sheep and goats. By vaccinating pregnant dams, the vaccine effectively curtails abortion rates caused by acute infection. Despite its success in reducing economic impact, the vaccine is limited by its inability to fully prevent cyst formation, the theoretical risk of virulence reversion associated with live-attenuated formulations, and a safety profile unsuitable for human use.

The genome-wide coverage (>99%) revealed in our study positions base editing as an ideal tool for high-throughput functional gene screens in *T. gondii*. What differs from traditional methods is that CRISPR knockout libraries require selection markers for each gene [34,35], whereas base editing can be performed in a marker-free manner, enabling pooled screening approaches similar to those used in mammalian cells [36]. Researchers can now systematically interrogate gene function without the complexity of constructing an individual *T. gondii* knockout strain.

Furthermore, the ability to introduce precise point mutations enables detailed structure-function studies and drug resistance modeling. For example, specific amino acid substitutions in drug target enzymes (e.g., DHFR-TS, enoyl-ACP reductase) can be introduced to validate resistance mechanisms identified in clinical isolates. This capability is particularly valuable given the limited arsenal of anti-toxoplasmosis drugs and the emergence of treatment failures in congenital toxoplasmosis.

We acknowledge certain limitations of the current system. Conventional BEs are largely constrained to C → T, A → G, and their complementary base transitions, and cannot efficiently mediate transversion mutations, precise insertions, or deletions [37]. Similar to other CRISPR-Cas9-based tools, base editing systems are constrained by their dependence on specific PAM sequences across different Cas protein variants, which restricts the targeting scope of current Cas proteins and limits the number of addressable genomic loci [29,32]. It should be acknowledged that episomal expression raises the risk of continuous nickase/deaminase expression, even independently of sgRNA, which may lead to continuous editing, potential off-target effects, or unintended phenotypic changes. BEs often exhibit bystander editing, in which multiple editable bases are present within the narrow activity window, leading to unwanted byproduct mutations alongside the desired edit. Although base editing substantially reduces byproducts associated with conventional CRISPR‑Cas9‑mediated DSBs, DNA and RNA off-target effects remain a central safety concern limiting clinical translation [38–40]. RNA off-target editing was not experimentally assessed in this study, given its importance for base editing safety, future studies should employ RNA-seq coupled with stringent bioinformatics pipelines, or engineered deaminase variants with reduced RNA activity [41], to comprehensively assess and minimize RNA off-target effects in *T. gondii*. In addition, since only ROP18 has been functionally validated, systematic testing across additional *T. gondii* genes is required to define the system’s scope of application and potential limitations.

Future efforts should focus on optimizing editor specificity and expanding the targeting scope through engineered Cas variants with relaxed PAM requirements, such as SpRY and SpG Cas9 variants [42] (recognizing NRN and NGN, respectively), to broaden the spectrum of targetable *T. gondii* genomic loci. Engineering both guide RNAs and the deaminase enzyme to minimize bystander editing without sacrificing activity, such as V28C [43]. Screening and employing endogenous high‑activity strong promoters of *T. gondii* (e.g., constitutive promoters highly expressed in tachyzoites and bradyzoite‑specific promoters) to drive the expression of ABE/CBE editors can elevate the transcriptional level and sustained expression stability of editors in parasites, thereby enhancing base editing efficiency at the source. According to the codon usage bias and translatome characteristics of the *T. gondii* genome, full‑sequence codon optimization is performed on core components such as the Cas9 nickase and deaminase to eliminate rare codons and match the translation machinery of Apicomplexan parasites, which significantly improves the translation efficiency and correct folding of editor proteins. Moreover, the development of drug-inducible base-editing systems under the control of regulated promoters will enable temporal control of editing activity, thereby reducing potential cytotoxicity associated with sustained expression of editing components.

Prime editing (PE) is a versatile and precise genome-editing technology that leverages a fusion of a Cas9 nickase, a reverse transcriptase, and a PE guide RNA (pegRNA) to mediate a wide range of genomic modifications. Unlike BEs, which are limited to single-base transitions, PE can achieve precise transversion mutations, small insertions (up to ~40 bp), deletions (up to ~80 bp), and single-base substitutions [44]. This editing strategy avoids DSBs and their associated off-target effects and byproducts, while offering greater flexibility in editing outcomes and higher precision. Notably, PE has not yet been fully developed or applied in *T. gondii*, making it a novel and highly promising avenue for genetic manipulation tools in this parasite. Introducing and optimizing a PE system tailored to *T. gondii*’s unique genomic context, cellular machinery, and transcription profiles will fill the gap in current editing technologies, overcoming the limitations of conventional BEs and enabling more versatile, precise genetic manipulation—an essential step forward for functional genomics research, parasite biology studies, and the development of novel anti-toxoplasmosis strategies.

Beyond *T. gondii*, the methodological frameworks and optimization strategies established in this work are readily adaptable to other apicomplexan parasites, including *Plasmodium spp*., *Cryptosporidium spp.*, and *Babesia spp.*, all of which remain genetically intractable due to limitations in existing genetic manipulation tools. Thus, the successful implementation of base editing in *T. gondii* establishes a critical proof of principle for precision genome engineering across the entire phylum Apicomplexa, with the potential to advance research and therapeutic development for multiple devastating human and veterinary pathogens.

In summary, we have validated base editing as a precise and efficient genetic tool that provides a powerful alternative to conventional methods, serving as an essential platform for basic research, drug discovery, and vaccine development, and thereby laying the foundation for precise genetic manipulation to accelerate basic and translational research in *T. gondii* (Fig 4I).

## Supporting information

S1 Table The sgRNAs used in this study were selected after screening and validation. (XLSX)

S2 Table The primers used in this study were all specific primers selected after validation. (XLSX)

S3 Table The off-target sequences. (XLSX)

S4 Table Editing efficiencies and sequencing depth. (XLSX)

S5 Table Complete set of i-STOP targetable sites across the entire genome of the *T. gondii* ME49 strain. (XLSX)

S6 Table Complete set of i-Silence targetable sites across the entire genome of the *T. gondii* ME49 strain. (XLSX)

S1 Raw Gel Original images for blots and gels. (PDF)
