# Supplementary material for: Base editing-mediated precision genome engineering in Toxoplasma gondii
Source: PLoS Negl Trop Dis. 2026 Nov 13;20(11):e0014864. doi: 10.1371/journal.pntd.0014864 (PMC13766282; doi:10.1371/journal.pntd.0014864)
Supplement: Supplementary file 1 [file pntd.0014864.s007.pdf]

Fig 1B

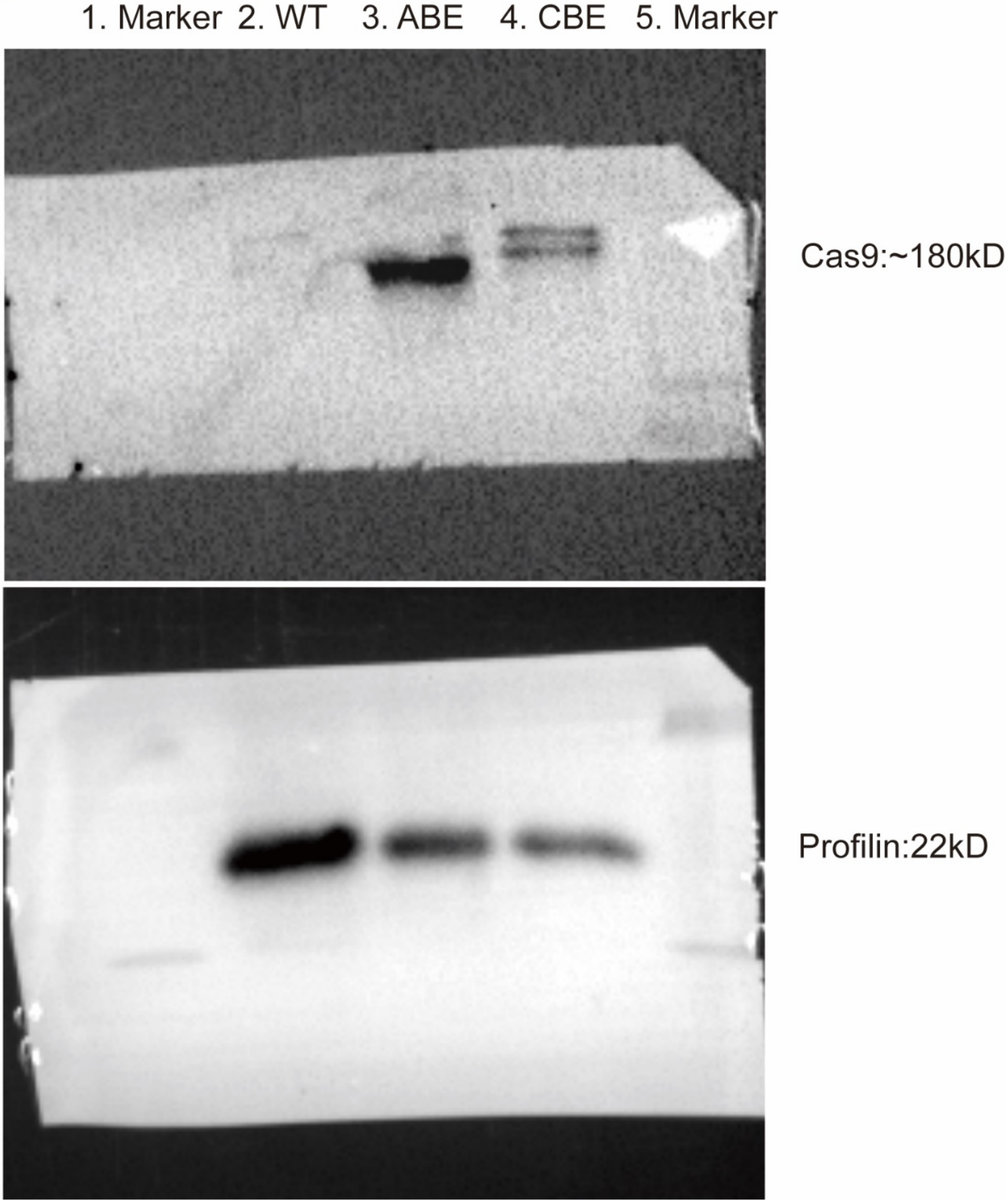

Fig 2B

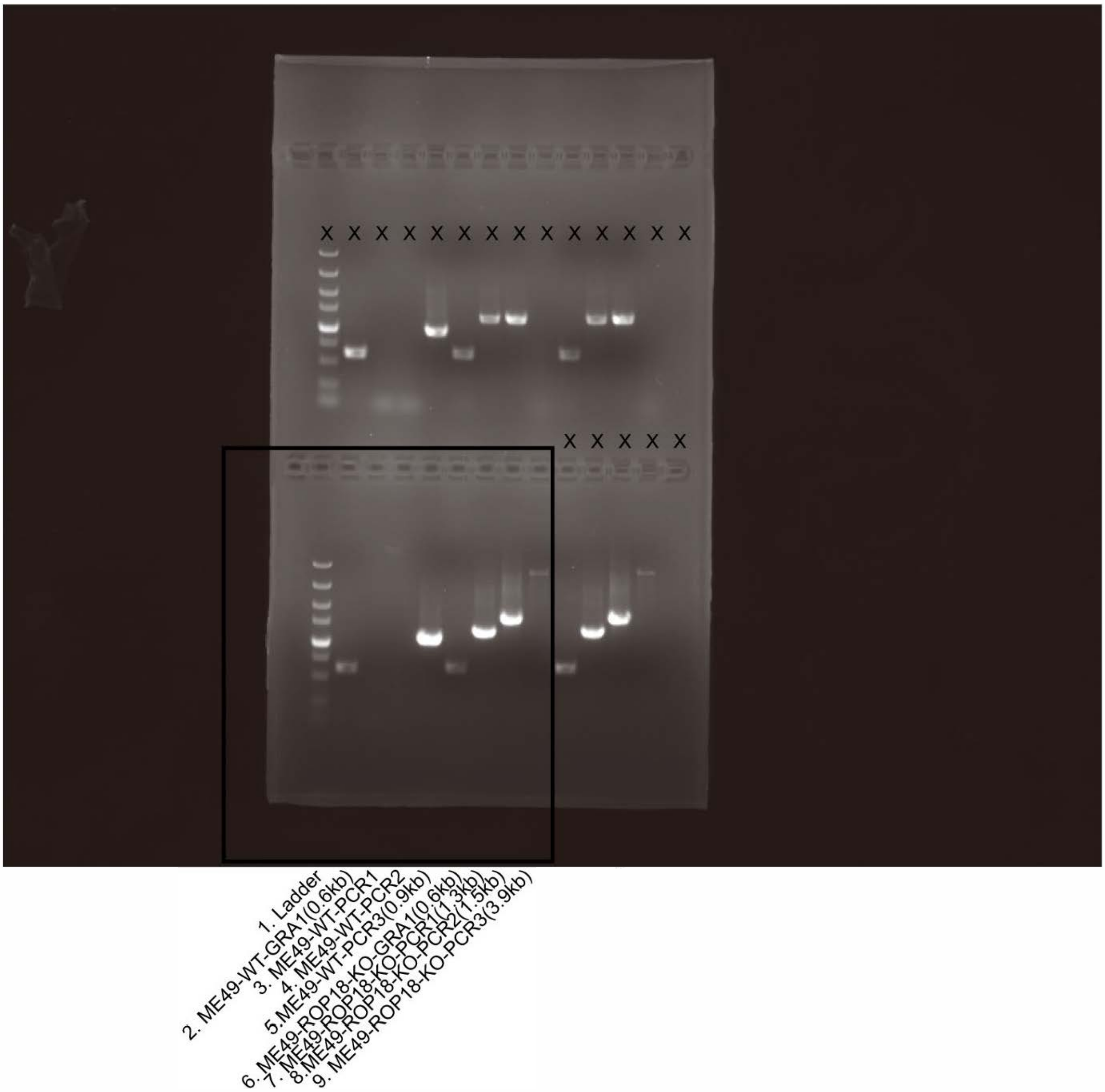
X X X X X X X X X X X X X X
X X X X X
1. Ladder
2. ME49-WT-GRA1(0.6kb)
3. ME49-WT-PCR1
4. ME49-WT-PCR2
5.ME49-WT-PCR3(0.9kb)
6. ME49-ROP18-KO-GRA1(0.6kb)
7. ME49-ROP18-KO-PCR1(1.3kb)
8.ME49-ROP18-KO-PCR2(1.5kb)
9. ME49-ROP18-KO-PCR3(3.9kb)

Fig 2C

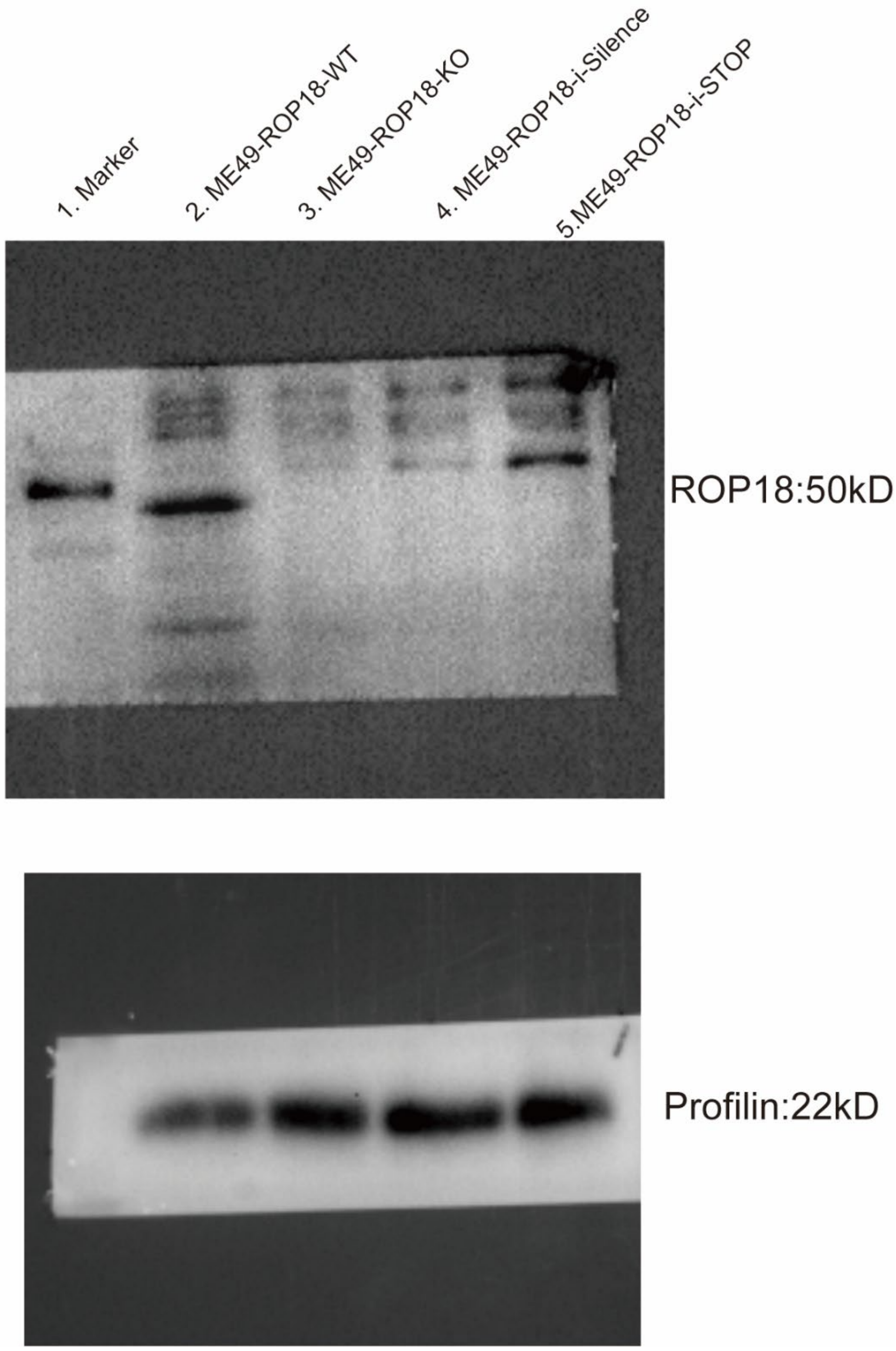

1. Marker
2. ME49-ROP18-WT
3. ME49-ROP18-KO
4. ME49-ROP18-i-Silence
5.ME49-ROP18-i-STOP
ROP18:50kD
Profilin:22kD
